# Divalent EuRh_2_Si_2_ as a reference for the Luttinger theorem and antiferromagnetism in trivalent heavy-fermion YbRh_2_Si_2_

**DOI:** 10.1038/s41467-019-08688-y

**Published:** 2019-02-15

**Authors:** M. Güttler, A. Generalov, S. I. Fujimori, K. Kummer, A. Chikina, S. Seiro, S. Danzenbächer, Yu. M. Koroteev, E. V. Chulkov, M. Radovic, M. Shi, N. C. Plumb, C. Laubschat, J. W. Allen, C. Krellner, C. Geibel, D. V. Vyalikh

**Affiliations:** 1 Institut für Festkörper- und Materialphysik, Technische Universität Dresden, D-01062 Dresden, Germany; 2 MAX IV Laboratory, Lund University, Box 118, 22100 Lund, Sweden; 3 Materials Sciences Research Center, Japan Atomic Energy Agency, Sayo, Hyogo, 679-5148 Japan; 4 European Synchrotron Radiation Facility, 71 Avenue des Martyrs, 38043 Grenoble, France; 5 Swiss Light Source and Swiss FEL, Paul Scherrer Institute, CH-5232 Villigen-PSI, Switzerland; 6 IFW Dresden, Helmholtzstr. 20, 01069 Dresden, Germany; 7 Max-Planck-Institut für Chemische Physik fester Stoffe, 01187 Dresden, Germany; 8 Tomsk State University, Lenina Av., 36, Tomsk, Russia 634050; 9 Institute of Strength Physics and Materials Science, RAS, Tomsk, Russia 634055; 10 Centro de Física de Materiales CFM-MPC and Centro Mixto CSIC-UPV/EHU, 20018 San Sebastián/Donostia, Spain; 11 Donostia International Physics Center (DIPC), 20080 San Sebastian, Spain; 12 Saint Petersburg State University, Saint Petersburg, Russia 198504; 13 Randall Laboratory, University of Michigan, 450 Church St, Ann Arbor, MI 48109-1040 USA; 14 Kristall- und Materiallabor, Physikalisches Institut, Goethe-Universität Frankfurt, Max-von-Laue Strasse 1, 60438 Frankfurt am Main, Germany; 15 IKERBASQUE, Basque Foundation for Science, 48011 Bilbao, Spain

## Abstract

Application of the Luttinger theorem to the Kondo lattice YbRh_2_Si_2_ suggests that its large 4*f*-derived Fermi surface (FS) in the paramagnetic (PM) regime should be similar in shape and volume to that of the divalent local-moment antiferromagnet (AFM) EuRh_2_Si_2_ in its PM regime. Here we show by angle-resolved photoemission spectroscopy that paramagnetic EuRh_2_Si_2_ has a large FS essentially similar to the one seen in YbRh_2_Si_2_ down to 1 K. In EuRh_2_Si_2_ the onset of AFM order below 24.5 K induces an extensive fragmentation of the FS due to Brillouin zone folding, intersection and resulting hybridization of the Fermi-surface sheets. Our results on EuRh_2_Si_2_ indicate that the formation of the AFM state in YbRh_2_Si_2_ is very likely also connected with similar changes in the FS, which have to be taken into account in the controversial analysis and discussion of anomalies observed at the quantum critical point in this system.

## Abstract (web summary)

Heavy-fermion materials have unusual electronic behavior due to a dual localized-itinerant character of 4*f* electrons. Here, by studying divalent EuRh_2_Si_2_, the authors gain insight into the electronic states of the trivalent heavy fermion system YbRh_2_Si_2_ and show that it experimentally demonstrates Luttinger’s theorem.

## Introduction

The determination of Fermi surfaces (FSs) plays a central role in understanding the many-body physics of strongly correlated metals. This is because most of the exciting phenomena in these systems are governed by thermally excited quasiparticles confined to a narrow window around the Fermi energy (*E*_F_)^1–5^. Furthermore, FSs are often the key to discriminating between rivaling theoretical models describing particular correlated metals^1,5^. For periodic Kondo lattice (KL) materials, where local orbital electrons both carry a magnetic moment and are coupled to conduction bands, the central issue is the extent to which the moment bearing electrons are included in the FS and the way in which this might be accomplished. A fundamental guiding theoretical principle was established by the seminal realization of R. M. Martin^6,7^ that the Luttinger FS sum rule^7^ can be applied even to such strongly correlated systems.

**Fig. 1:**
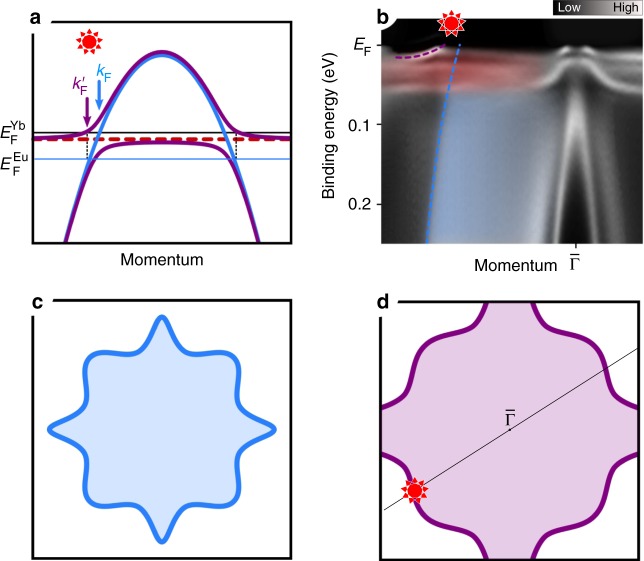
Formation of the large Fermi surface in a Kondo lattice. **a** Schematic hybridization model for Yb case: the localized and renormalized level of the 4*f* hole (dashed red line) hybridizes with a light hole-like conduction band (blue) and forms a heavy-quasiparticle band (purple) right below the Fermi level, *E*_F_. The Fermi vector shifts from *k*_F_ to *k*′_F_. The red sun symbol indicates the region of *f-d* hybridization. **b** ARPES band map of YbRh_2_Si_2_ (compare ref. ^9^). The Brillouin zone cut corresponds to the black line shown in **d**. The crystal-field-split Yb 4*f* bands (highlighted in red) hybridize with a bunch of projected bulk bands (shaded in blue) forming a heavy hole-like quasi-particle band at low temperature and shifting the Fermi vector outwards. **c**, **d** Schematic representation of a small and large Fermi surface: due to the formation of the Kondo lattice and the Luttinger theorem, the small hole pocket in **c** transforms into a large hole pocket in **d**, which incorporates now the additional 4*f* hole

The sum rule states that the FS volume is conserved in the presence of interactions so long as the interactions do not produce a phase transition. In this context, the essence of the sum rule is that if the local orbital magnetic moments are quenched, for example by the Kondo effect, then the underlying local orbital electrons must be counted in the FS. If the moments are not quenched, for example in a magnetically ordered state, the underlying electrons are excluded from the FS. For the KL, the sum rule is satisfied by the formation of heavy quasi-particle states at the Fermi energy, the analog of the Kondo resonance for a local orbital impurity site. The behavior along the line of the Fermi energy labeled *E*_F_^Yb^ in Fig. 1a shows, for the hole situation that will be relevant for this paper, how hybridization of the local orbital quasiparticle into the conduction bands enlarges the hole-like Fermi surface size from *k*_F_ to *k*′_F_ to count the local orbital hole. These two situations are now known as the small and large Fermi surfaces, respectively^8,9^.

Even though the initial theoretical realization is now many years in the past, clear experimental demonstrations of the both Luttinger theorem and its workings for KL materials are still very few. Measurements with the de Haas van Alphen (dHvA) technique have provided some observations of both the small and large FS^10^. But for angle resolved photoemission spectroscopy (ARPES) the large FS has been observed thus far only in YbRh_2_Si_2_^8,9^, while for another paradigm system CeCoIn_5_ the situation remains problematic and controversial^11,12^. This paucity is particularly unfortunate because ARPES is the FS technique most evocative for this concept, in that it allows to see directly both the Fermi surface and the underlying dispersing quasi-particles that define it.

Here, we present an ARPES study which reveals both the workings of the Luttinger sum rule and also another very general FS principle pointed out by Müller–Hartmann^13^, that if the single particle self energy for a dispersing quasi-particle is independent of the crystal momentum **k**, as is highly plausible for the 4*f* electrons of a rare-earth material, not only the FS size but also its shape is conserved for symmetry preserving interactions. These two concepts together are the reason why the FSs found in density functional theory (DFT) can often be in good agreement with experiment for rare-earth materials.

As explained further below, we compare two isoelectronic and isostructural rare-earth materials, YbRh_2_Si_2_ (YRS) and EuRh_2_Si_2_ (ERS), both in the paramagnetic (PM) state, the former where the FS encloses one strongly correlated 4*f* hole and *N* weakly correlated conduction band holes, and the latter where the FS encloses *N* *+* *1* weakly correlated holes, i.e. the same total hole count for both, implying a large FS for both. The two situations are illustrated in Fig. 1a by the *E*_F_ lines labeled Yb and Eu, respectively. Our very pretty result is that, both in ARPES and DFT, the FSs of both materials are essentially similar in size and shape, even though the former has the dispersion of a heavy mass and the latter has a light mass.

## Results

### Strongly correlated YbRh_2_Si_2_ and weakly correlated EuRh_2_Si_2_

Heavily studied YRS^14–17^ is a canonical heavy-fermion KL material, with antiferromagnetic (AFM) order below a Néel temperature *T*_N_ of 70 mK and superconductivity below 2 mK^17^. The AFM transition lies very close to a quantum-critical point (QCP) induced by a tiny magnetic field of 60 mT applied along the basal plane, that suppresses the magnetic order. The Yb valence ν is near 3^+^ which gives one hole in the 4*f* shell. If magnetic moments are Kondo quenched in the low temperature paramagnetic phase just above *T*_N_, we would expect this hole to be included in the FS. As will be seen in more detail below, DFT shows that the generic RERh_2_Si_2_ (RE = rare earth) FS consists of two sheets called the Doughnut (*D)* and the Jungle-gym (*J*)^8–10,18^. The Jungle-gym forms a largely interconnected Fermi surface sheet across the whole **k**-space, whereas the Doughnut consists of a square-like, large hole pocket around the Z-point of 3D Brillouin zone (BZ).

The topology of the Doughnut can be used to distinguish the large (itinerant 4*f* hole) and the small (localized 4*f* hole) FSs^8–10^, via the size of its necks which, respectively, do or do not extend into neighboring BZs, as shown schematically in Fig. 1c, d. Our past ARPES work for YRS clearly shows the large FS of Fig. 1d down to 800 mK^8^. The YRS ARPES spectrum of Fig. 1b shows the heavy 4*f* quasi-particle and the itinerant band states dispersing across *E*_F_ marked with colors corresponding to the schematic of Fig. 1a. As a calibration, the small FS of Fig. 1c has been conclusively verified by ARPES and DFT for the stable trivalent, local moment AFM, weakly correlated, isoelectronic reference system YbCo_2_Si_2_^8,19^, see also Supplementary Note 1.

ERS, the material studied in this paper, is also a weakly correlated, local moment AFM isoelectronic reference system^20–22^, but with a valency of 2^+^ it is divalent. Thus, in accordance with the Luttinger sum rule, ERS in its paramagnetic phase should possess the large FS, and in the first part of the paper we will show that this is indeed the case. Further, not only can the electronic structure and Fermi surface for the AFM phase of ERS be calculated easily with DFT, but also the transition at *T*_N_ = 24.5 K can be explored conveniently with ARPES. In the second part of the paper we present the respective DFT and ARPES results for the AFM phase and, finally, we use these data to discuss possible changes in the electronic structure of YRS in its AFM phase below 70 mK and the possible implications for its QCP behavior.

### Paramagnetic phase of EuRh2Si2 from DFT and UV-ARPES

**Fig. 2:**
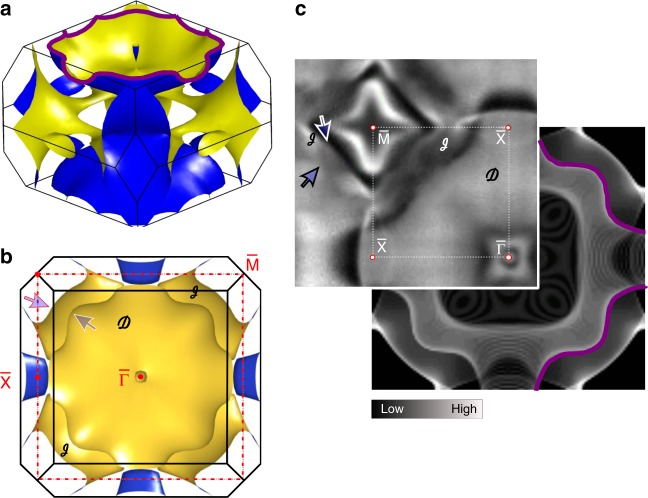
Fermi surface for the paramagnetic EuRh_2_Si_2_. Three-dimensional representation of the bulk Fermi surface with the Doughnut and Jungle-gym sheets in a side **a** and top **b** views. The red dotted line in **b** depicts the surface BZ. **c** ARPES-derived Fermi-surface measured with 45-eV photons combined with the projected bands from bulk band-structure calculations

The DFT-derived FS of ERS in the PM phase is shown in Fig. 2a, b as a side and top view, respectively. It very closely resembles the one established for YRS in the Kondo PM regime and both the Doughnut and the Jungle-gym can clearly be seen. The center and inset panels of Fig. 2c compare, respectively, DFT and UV-ARPES data for PM ERS, taken with 45 eV photon energy. For this photon energy and the resulting photoelectron kinetic energy, the photoelectron has a very small relaxation time and a short mean free path. In consequence^23^ our UV-ARPES data is both surface sensitive and *k*_*z*_ broadened so that the bulk electronic structure is mostly **k**-summed along *k*_*z*_. Therefore the DFT-derived electronic structure is shown projected along *k*_*z*_ onto the 2D surface BZ, which is depicted as a dashed-red line in Fig. 2b. High symmetry points of the surface BZ are marked in both panels with overlined symbols. The large Doughnut sheet can be well distinguished and is seen as a homogenous gray area. The projected Jungle-gym is weakly visible in the first BZ, but due to photoemission cross-section effects it is much more pronounced in the second BZ. Those parts that build the border of the projected gap around the ${\bar{\mathrm M}}$-point and are not masked by the Doughnut, are indicated by arrows. In addition to these features predicted by the bulk band-structure calculations, sharp spectral patterns around the $\overline \Gamma$-point as well as the diamond-shaped feature around the ${\bar{\mathrm M}}$-point result from surface resonances and Shockley surface states. The Shockley state has been discussed in detail in earlier works^24–26^. Because of their surface origin these features are obviously missing in the calculated bulk bands in Fig. 2, but can be reproduced and identified with the help of slab band-structure calculations and thus be well separated from bulk contributions to the spectra.

The projected Fermi surface excluding the surface-related states is in perfect agreement with the calculated 3D Fermi surface and can thus be attributed to the projected bulk band structure in the PM phase. This result, together with the recently presented large Fermi surface for YRS^8,9^ also derived by ARPES, shows nicely and clearly the working of the Luttinger theorem for the PM phases of YRS and ERS (see also Supplementary Note 2 and Supplementary Figure 1) and leaves us with a solid starting point for exploring in more detail how the Fermi surface will be modified in the AFM phase of ERS.

**Fig. 3:**
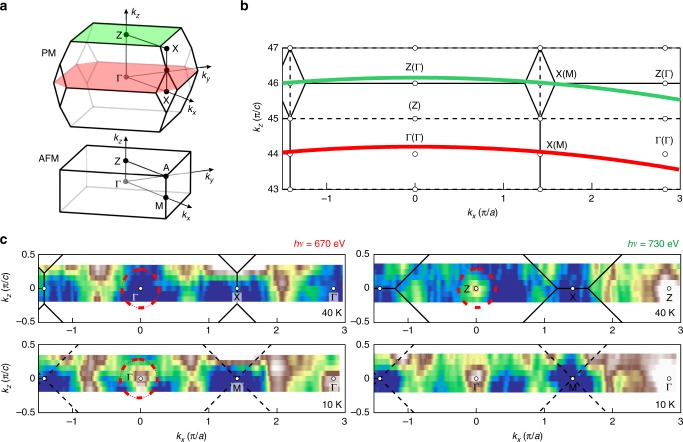
PM-to-AFM transition in EuRh_2_Si_2_ measured by soft X-ray ARPES. **a** Brillouin zones of body-centered (PM) and simple tetragonal (AFM) EuRh_2_Si_2_. **b** Navigation through the 3D *k*-space. The solid (dashed) lines indicate the boarders of the Brillouin zones along with corresponding high symmetry points in the PM (AFM) phase. The photoemission measurement arcs, probing the crystal momenta close to the Γ- and Z- points are shown in red (*hν* = 670 eV) and green (*hν* = 730 eV), respectively. **c** Fermi surface sections derived from soft X-ray ARPES performed at 40 K (for the PM phase) and at 10 K (for the AFM phase)

For the AFM phase of ERS, an incommensurate magnetic order with a propagation vector **Q** = (0, 0, *τ*) with *τ* ≈ 0.79 has been established experimentally^24^. If we approximate the propagation vector close to the sample surface with *τ* = 1, the unit cell in real space is doubled due to the lowered symmetry and the BZ is halved changing from a truncated octahedron to a simple tetrahedron upon entering the AFM phase, as shown in Fig. 3a. Correspondingly, this transition should be accompanied by a back-folding of electron bands into the halved BZ, i.e. the Z-point will be folded onto the Γ-point and the electronic structure at Z and Γ of the PM phase should become equivalent as two Γ points of the AFM phase. Observation of this basic change requires resolution along *k*_*z*_, which precludes using the *k*_*z*_ broadened UV-ARPES spectra. Therefore we performed truly bulk sensitive soft X-ray ARPES measurements.

### Soft X-ray ARPES on EuRh_2_Si_2_

At such high photon energies, the probing depth is quite high compared to that of UV-ARPES, the probability to excite surface-related states is significantly reduced, and we can study the bulk *k*_*z*_ dependence (a complete *k*_*x*_ **-** *k*_*z*_ map obtained by soft X-ray ARPES is presented in Supplementary Figure 2 and discussed in Supplementary Note 3). The energy resolution is reduced but nonetheless adequate for the purpose at hand. We used photon energies of 670 eV and 730 eV, which probe the electronic structure along the measurement arcs shown in Fig. 3b. These provide planes of approximately constant *k*_*z*_ near the Γ- and Z- point of the paramagnetic BZ. In Fig. 3c, we compare ARPES-derived Fermi surface maps obtained in the PM phase at *T* *=* 40 K and the AFM phase at *T* *=* 10 K, see also Supplementary Figure 3 and Supplementary Note 4. As expected, in the PM phase we see distinctly different spectra in the regions of the inequivalent Γ- and Z- points. In particular, a hole-like pocket can be seen around Z, whereas no such pocket exists around Γ. Upon cooling the system down to 10 K below the Néel temperature, a similar hole-like band as that around Z now appears around the Γ-point, where the intensity was previously depleted. At the same time, the hole pocket around Z seems to be unaffected by the AFM transition. The appearance of spectral weight around Γ in the AFM phase, which in the PM phase could be seen exclusively around Z, is fully consistent with the change of the BZ and the resulting back-folding of electronic bands.

### Antiferromagnetic phase of EuRh_2_Si_2_ from DFT and UV-ARPES

**Fig. 4:**
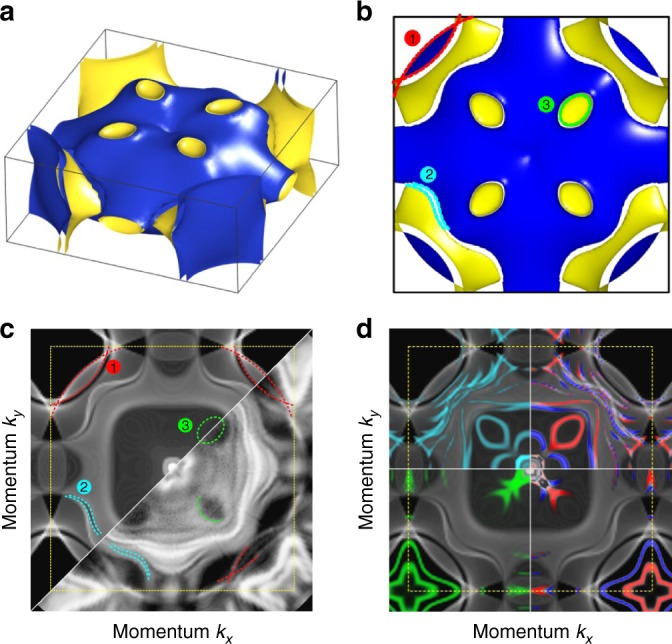
Fermi surface of AFM-ordered EuRh_2_Si_2_ derived by UV-ARPES and DFT calculations. **a** 3D view and **b** top view of the calculated FS along *k*_*z*_. **c** Comparison of the projected bulk FS (top left panel) and the ARPES-derived FS (bottom right panel) taken at 10 K. The yellow dashed line depicts the border of the Brillouin zone. **d** Additionally to the projected bulk FS, the surface states and surface resonances have been extracted from slab calculations for Eu-termination (cyan, top left panel) and Si-termination (green, bottom left). The spin polarization of the termination-specific surface-related states is indicated in red and blue on the right side

Having experimentally established the back folding of electron bands along the Γ - Z direction across the AFM transition, we turn now to the results of our band structure calculations to analyze in more detail the changes of the electronic structure upon entering the AFM phase. The DFT-derived FS is shown in Fig. 4a, b as a side and top view, respectively. The Doughnut and parts of the Jungle-gym are folded into the new BZ. The Doughnut is folded onto the Γ-point along with the previous Z-point of the PM phase. The new Z-plane, which lies halfway between the Γ- and Z-points of the PM structure, cuts the folded Doughnut slightly, resulting in small oval hole pockets labeled (3) in Fig. 4b and marked in green (see Supplementary Figure 4). These pockets are oriented towards the corners of the simple-tetragonal BZ and are a further important indicator for the folded FS in the AFM phase. The large Jungle-gym then hybridizes with the Doughnut, splitting up into two distinct sheets. The resulting splitting of the FS is labeled (2) in Fig. 4b and marked in cyan. The outermost sheet enclosing the BZ corners is labeled (1) in Fig. 4b and marked in red. This banana-shaped feature results from the folding of the Jungle-gym onto itself, splitting off due to the exchange interaction to form long tubes with almost parallel surfaces along *k*_*z*_. Similarly, the outer of the two sheets of feature (2) also forms long, but warped, tubes along *k*_*z*_. As a general conclusion, the bulk DFT calculation predicts a strong fragmentation of the PM FS upon the AFM transition.

We now compare the band structure calculation to the FS experimentally obtained by UV-ARPES at a temperature of 10 K, sufficiently below the Néel temperature. As with the PM phase we must account for the *k*_*z*_ broadening of bulk features and the sensitivity to surface features. We consider the bulk features first and show in the upper left part of Fig. 4c the calculated bulk FS projected along *k*_*z*_. As labeled, it still shows nicely the same three key features already called out in Fig. 4b. The gap labeled (2) lies within the projected Doughnut close to the borders. The ARPES-derived FS in the lower right half of Fig. 4c shows these same three bulk features, as indicated by dashed lines with the same color coding. Although the ARPES intensity of the PM phase Jungle-gym is quite low in the first BZ, the borders of the outer FS sheet, which coincide with the banana-shaped feature (1), are clearly visible. The ARPES-derived FS also shows the gap labeled (2) and additionally there are four oval gap-like features which appear exactly where the oval hole pockets (3) are expected.

Considering next the surface, by comparing the ARPES data to slab DFT calculations we find that all remaining features can be understood as a superposition of spectra from both Si and Eu terminations present together on our measured surfaces. In our slab DFT calculations, a supercell consisting of a thick slab of four conventional unit cells (32 atomic layers) surrounded by empty space mimics the two surfaces of the material with several bulk layers in between and enables us to study the surface modifications of the band structure. In Fig. 4d, we overlaid the projected bulk bands (shaded in gray) with the surface states and surface resonances of the FS obtained from our slab calculation (colored). To identify the surface character, only states with a predominant contribution in the topmost four atomic layers are extracted from the overall slab band structure. The two segments at the top colored cyan and red/blue are calculated for the Eu-terminated half of the slab, the two segments at the bottom colored green and red/blue are obtained from the Si-terminated part. The cyan and green states indicate surface states and resonances for the respective terminations. The red and blue coloring on the right side additionally points out the spin polarization of these states.

We have already identified in the PM phase UV-ARPES spectra a pronounced well known Shockley state around the ${\bar{\mathrm M}}$-point. We see that this strictly two-dimensional Shockley state splits off from the border of the projected bulk band gap, occurs only for Si-termination, and is strongly spin split due to exchange interactions with the ferromagnetically polarized Eu 4*f* moments, both in the calculated AFM FS and in the AFM ARPES data. This characteristic fingerprint thus clearly indicates the presence of Si-terminations in UV-ARPES.

For Eu-termination there are a bunch of surface resonances that arise from bulk states, are pinned to the topmost Eu surface layer, and are further spin split. These account nicely for what is perhaps the most striking and instructive feature in the UV-ARPES derived FS, a dense bunch of bright and rather sharp states, which appear within the Doughnut following roughly its shape. Multiply repeated experiments convincingly indicate reproducibility of this spectral pattern. These states obviously cannot be observed in the PM phase (compare Fig. 2c) and are not present in the calculated bulk FS of the AFM phase.

## Discussion

What are the relevance and the implications of our observations on the AFM state of EuRh_2_Si_2_ for the system YbRh_2_Si_2_? In the debate on the nature of the field induced quantum critical point in YbRh_2_Si_2_, the evolution of the Fermi surface from the magnetically non-ordered state to the AFM state is a pivotal issue^15,16,27^. Most of the discussion has focused on the possibility of a change in the nature of the quasiparticle, while the effect of the magnetic ordering on its own has not been considered. However, the present observations on EuRh_2_Si_2_ show that magnetic ordering results in large changes in the Fermi surface through band folding and band splitting. These results cannot be taken over directly to YbRh_2_Si_2_ because the AFM structure of YbRh_2_Si_2_ is yet unknown. However, results on both static magnetic properties (e.g. magnetization) as well as dynamic magnetic properties (e.g. relaxation behavior) (see Supplementary Note 5) indicate that magnetic correlations in YbRh_2_Si_2_ are very similar to those in EuRh_2_Si_2_. In particular both display a strong competition between FM and AFM order. With the Fermi surfaces being so similar in the PM states of EuRh_2_Si_2_ and YbRh_2_Si_2_, and also a strong similarity in their magnetic correlations, EuRh_2_Si_2_ becomes a relevant model system for gauging the impact of AFM order on the Fermi surface of YbRh_2_Si_2_.

One concern with this approach might be that EuRh_2_Si_2_ is an RKKY mediated large local moment system whereas the extremely small moment of YbRh_2_Si_2_ strongly suggests that it should be regarded as a spin-density-wave (SDW) system (here SDW refers to its original sense as the ordering of delocalized electrons). However, for the relevance and resulting change of the Fermi surface, both cases are actually very similar. Because in RKKY exchange the transfer of information on the polarization from one 4*f* site to the next 4*f* site is exclusively due to conduction electrons, the generalized susceptibility χ(*q*) of the conduction electrons, and therefore e.g. nesting properties, is as much relevant for local moment ordering as for the SDW scenario. Accordingly, modifications of the band structure, at least on a qualitative level, should be very similar in both kinds of systems. Both our experimental ARPES results and our calculations show that for this Fermi surface, AFM order leads to significant changes, because of both the large amount of band folding and the large band splittings. Such large changes cannot be ignored in discussing the anomalies seen in a number of transport properties at the field induced quantum critical point (QCP) in YbRh_2_Si_2_^28^, of special significance because these anomalies are at the heart of a debate on the nature of this QCP^16,27^. For example, the Hall effect has received considerable attention^16,27^ and it surely must react to large Fermi surface changes. Thus, our results on EuRh_2_Si_2_ indicate that the formation of the AFM state in YbRh_2_Si_2_ very likely results in strong changes in the large Fermi surface. Within this picture, the Fermi surface changes would be due to the magnetic order and not to a “large to small” transition arising from Kondo breakdown. These changes could well be sufficient for understanding the anomalous properties observed at the QCP on entering the AFM state. Folding and splitting of the bands is a priori expected to affect the thermodynamic and transport properties only within the AFM state, but not the critical behavior in the paramagnetic regime above *T*_N_. However, within this scenario the unconventional critical behavior observed in the paramagnetic regime can e.g. be accounted for by interaction between fluctuations of the antiferromagnetic order parameter and critically renormalized electron quasiparticles^27^. More experimental studies of the properties of YbRh_2_Si_2_ in the ordered phase and corresponding microscopic theory making use of the down-folded Fermi surface structure are needed to further explore this electronic structure model. But it is a testable hypothesis.

In summary, we have studied the Fermi surfaces for both the PM and AFM phases of EuRh_2_Si_2_ using soft X-ray and UV-ARPES combined with ab initio full relativistic DFT band structure calculations. In the PM state we identify the bulk Fermi surface in EuRh_2_Si_2_ (with divalent Eu) as being very similar to that of the Kondo system YbRh_2_Si_2_ (with nearly trivalent Yb). This is a beautiful new experimental visualization of the working of the Luttinger sum rule. For PM Kondo systems it predicts that the *f* degree of freedom, in the case of trivalent Yb the 4*f* hole, despite its localized character, must be counted in the Fermi volume, leading to the same Fermi volume as for a homolog compound with a divalent rare earth. Furthermore, our ARPES data and our calculations demonstrate that the onset of AFM order leads to pronounced changes in the Fermi surfaces due to band folding and band splitting. The strong changes between the PM and AFM states calculated and observed for EuRh_2_Si_2_ indicate that the formation of the AFM state in YbRh_2_Si_2_ very likely also results in strong changes in its large Fermi surface. Therefore, such kinds of changes, as an alternative to changes in the quasi-particle due to Kondo breakdown, have to be considered in the controversial analysis and discussion of the origin of the transport properties observed on crossing the QCP into the ordered state in this system.

As a final note, an important instructive result of our work is the clear demonstration of different sets of spin-polarized states persisting on two distinct surface terminations of EuRh_2_Si_2_. The Si terminated surface reveals mainly itinerant magnetism confined to the surface due to the presence of Shockley surface states, which couple via exchange interaction with the Eu 4*f* moments in the fourth layer below the Si surface. However, the Eu-terminated surface exhibits mainly bulk electron states, which show strong resonant behavior at the Eu surface. Due to the exchange coupling with the topmost polarized Eu 4*f* moments these states become themselves spin-polarized in the vicinity of the surface. These results clearly show that a magnetically ordered hetero system may reveal curious and profoundly different magnetism at its surface depending on the constituting sorts of surface atoms which is challenging to predict and can be interesting for technological applications.

## Methods

### Experiment

UV-ARPES and Soft X-ray ARPES experiments were carried out at the Swiss Light Source (SIS X09LA instrument) and SPring-8 synchrotron radiation facility (Beamline BL23SU), respectively. The UV-ARPES experiments were discussed in details in^24^, while the Soft X-ray ARPES experiments were comprehensively presented in ref. ^29^. The single crystals of EuRh_2_Si_2_ were grown in indium flux as described in^20,21^.

### Theory

The full-relativistic band structure calculations have been performed with the FPLO code^30^, a density functional theory code based on the full-potential local orbital method, within the generalized gradient approximation (GGA)^31^. The Eu 4*f* basis states have been fixed to the experimentally found occupation (*n*_4*f*_ ≈ 7.0^32^, frozen core approximation) using either an unpolarized configuration in the paramagnetic phase or a local-moment scenario of 7 μ_B_ per Eu atom with ferromagnetic coupling within the Eu *ab*-planes and antiferromagnetic interaction along the *c*-axis in the AFM phase. Surface effects were simulated by an asymmetric slab with 32 atomic layers terminated on one side by Si and on the other side by Eu atoms. Further details on the computations can be found in^24,33^.

## Supplementary Information

Supplementary Information

## Data Availability

All relevant data are available from the corresponding author upon request.
