# Supplementary material for: Divalent EuRh2Si2 as a reference for the Luttinger theorem and antiferromagnetism in trivalent heavy-fermion YbRh2Si2
Source: Nat Commun. 2019 Feb 15;10:796. doi: 10.1038/s41467-019-08688-y (PMC6377675; doi:10.1038/s41467-019-08688-y)
Supplement: Supplementary file 1 — Supplementary Information [file 41467_2019_8688_MOESM1_ESM.docx]

*Supplementary Information for*

**Divalent EuRh_2_Si_2_ as a reference for the Luttinger theorem and antiferromagnetism in trivalent heavy-fermion YbRh_2_Si_2_**

M. Güttler et al.

**Supplementary Note 1:**

When Yb behaves as a local moment ion with a valency of 3^+^, the hole-like Fermi surface reveals a “small” size, while when the 4*f* orbitals start to hybridize with the itinerant states and the valence changes even slightly to 2.9^+^, the Fermi surface reveals a strong modification that is prominently reflected in the opening of the necks to the neighboring BZ. A comparison of the published Fermi surface of YbRh_2_Si_2_ derived by ARPES with the Doughnut Fermi surface sheets calculated by Zwicknagl [1] and Rourke et al. [2] reflecting the large Fermi surface, reveals that the hole-counting Fermi surface volume contains a full additional hole, i.e. the Yb 4*f* hole couples completely to the Fermi surface, although the valence changes only by 0.1.

The Fermi surface of a purely trivalent Yb system like YbCo_2_Si_2_ [3] or LuRh_2_Si_2_ [2] is substantially smaller. In stark contrast to the large Fermi surface, the small Fermi surface excluding the Kondo-coupled 4*f* hole has clearly closed necks, which do not reach the neighboring BZ. It is important to note, that a simple rigid-band-filling increase of the Fermi volume by only 0.1 holes would by far not be sufficient to open these necks. Thus, the size and width of the necks is an essential fingerprint of the large difference in Fermi surface volume between the two situations.

**Supplementary Note 2:**

The ARPES data taken from EuRh_2_Si_2_ with divalent Eu show that the Doughnut necks are widely opened. The hole-like topology of the Doughnut is immediately evident from the dispersion of the bands constituting this Fermi surface sheet. In Supplementary Fig. 1, we present a comparison of ARPES-derived band maps along the $\bar{M}-\bar{X}-\bar{M}$direction taken from YbRh_2_Si_2_ (at T=11 K) and from EuRh_2_Si_2_ (T=34 K, PM phase) and demonstrate the derived widths of the necks in **k**-space. We found from our analysis that the neck width in both compounds differs by only about 15 %. Therefore we can conclude that the neck widths are indeed rather similar, keeping in mind the slightly different lattice parameters and therefore the different BZ sizes, inner potentials, temperature of the experiments etc. We thus concluded that the size of the Fermi surfaces is very similar and both Fermi volumes contain the same number of carriers. This is an unambiguous proof of the Luttinger theorem since compared to trivalent systems EuRh_2_Si_2_ adds a weakly correlated hole to the Fermi surface, while YbRh_2_Si_2_ in the Kondo regime features a strongly correlated hole, but the total number of holes is the same.

**
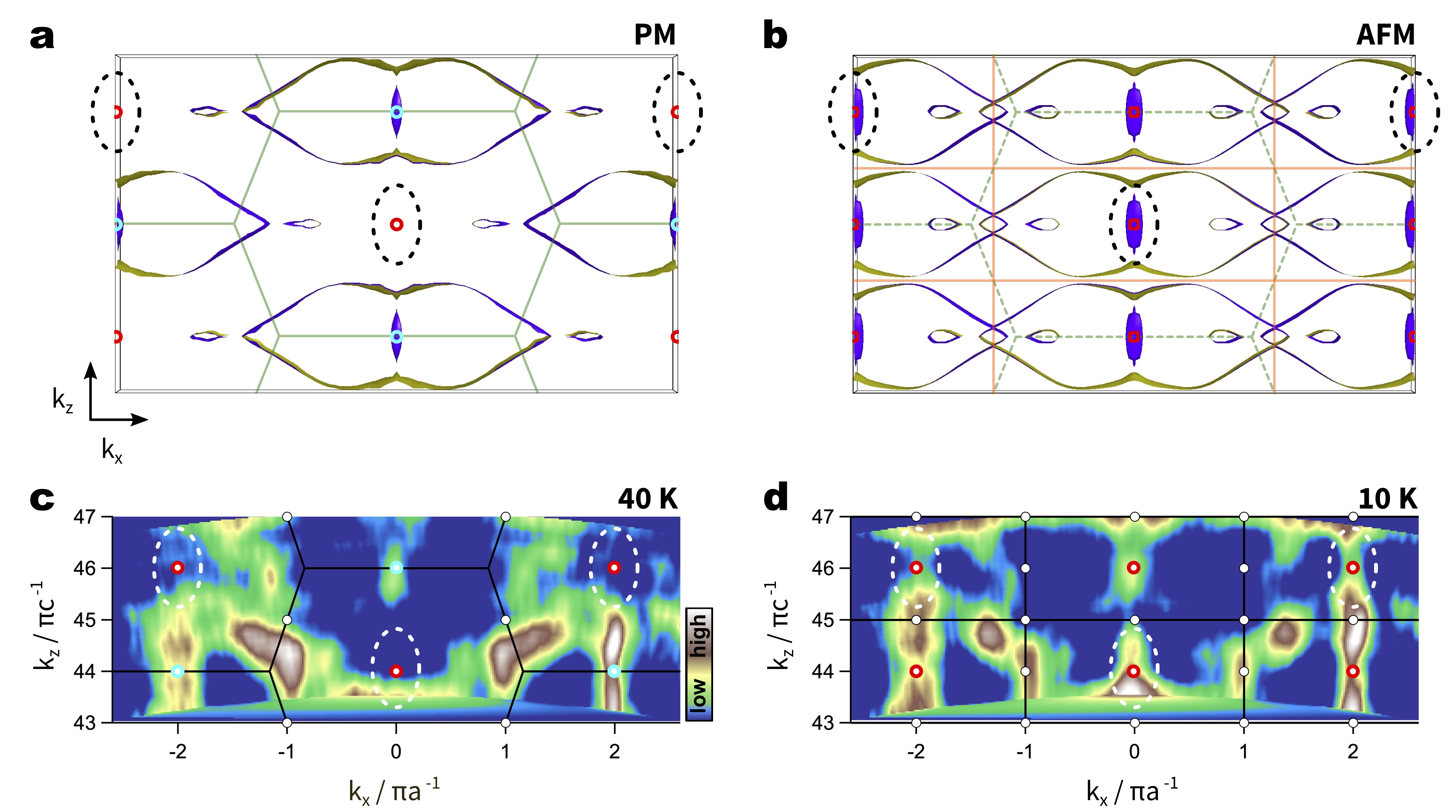
**

**Supplementary Figure 2:** 2D slices from the calculated 3D Fermi surfaces in the k_x_ – k_z_ plane along (a) Γ – M for the PM and (b) Γ – X for the AFM phase, respectively. See Fig. 3a in the manuscript for labeling of the high-symmetry points. Green (orange) lines represent the Brillouin zone borders in the PM (AFM) phase. The small red (cyan) circles in panels (a) to (d) denote Γ-(Z-) points. In panels (c) and (d), the Fermi surface in the k_x_ – k_z_ plane obtained by soft X-ray ARPES for photon energies between 650 and 760 eV is shown for (c) 40 K along Γ – M and for (d) 10 K along Γ – X. White/black dotted ovals in all panels indicate the location of the cigar-shaped hole pocket, which appears solely around the Z-point and is therefore missing around the Γ- point in the PM phase, while it is folded onto the Γ- point in the AFM phase.

**
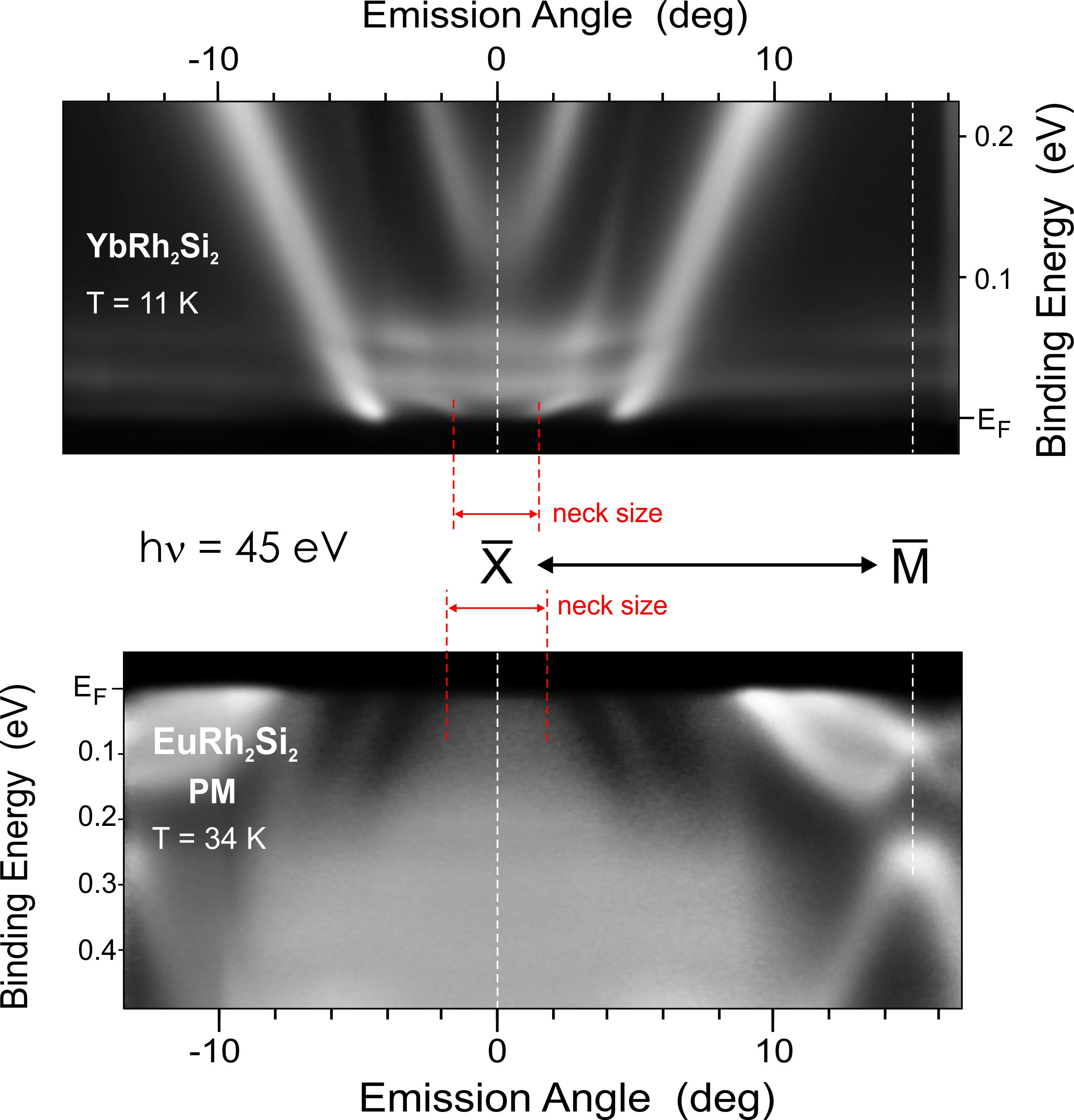
**

**Supplementary Figure 1**: ARPES derived band maps for YbRh_2_Si_2_ and EuRh_2_Si_2_ taken at 11 K and 34 K, respectively. The derived width of the neck in a **k**-space is demonstrated by the red dotted lines for both considered materials.

**Supplementary Note 3:**

In Supplementary Figure 2, we present the Fermi surface of EuRh_2_Si_2_ obtained by soft X-ray ARPES in the *k_x_ – k_z_* plane along Γ – M(X) in the PM (AFM) phases. For comparison, the calculated Fermi surface in the same **k**-space plane is depicted in panels (a) and (b). In the calculation, the largest Fermi surface sheets around the Z-points in the PM phase reaching into neighboring Brillouin zones can be identified as the Doughnut sheets. Additionally, narrow cigar-shaped hole pockets can be seen at the Z-points in the PM phase, while they are missing at the Γ-point. Upon entering the AFM phase, the Fermi surface including the cigar-shaped hole pockets gets folded onto the Γ-point. Similarly, the experimental data for paramagnetic EuRh_2_Si_2_ in panel (c) reveals a narrow vertical spectral feature at the Z-point, which is missing (inside the white dashed ovals) at the Γ-point. In the ordered phase, similar elongated features appear at Γ inside the dashed ovals and can be attributed to a folding of the cigar hole pocket. This finding is in full accordance to the data presented in Figures 3(c) in the manuscript and Supplementary Figures 2(a and b).


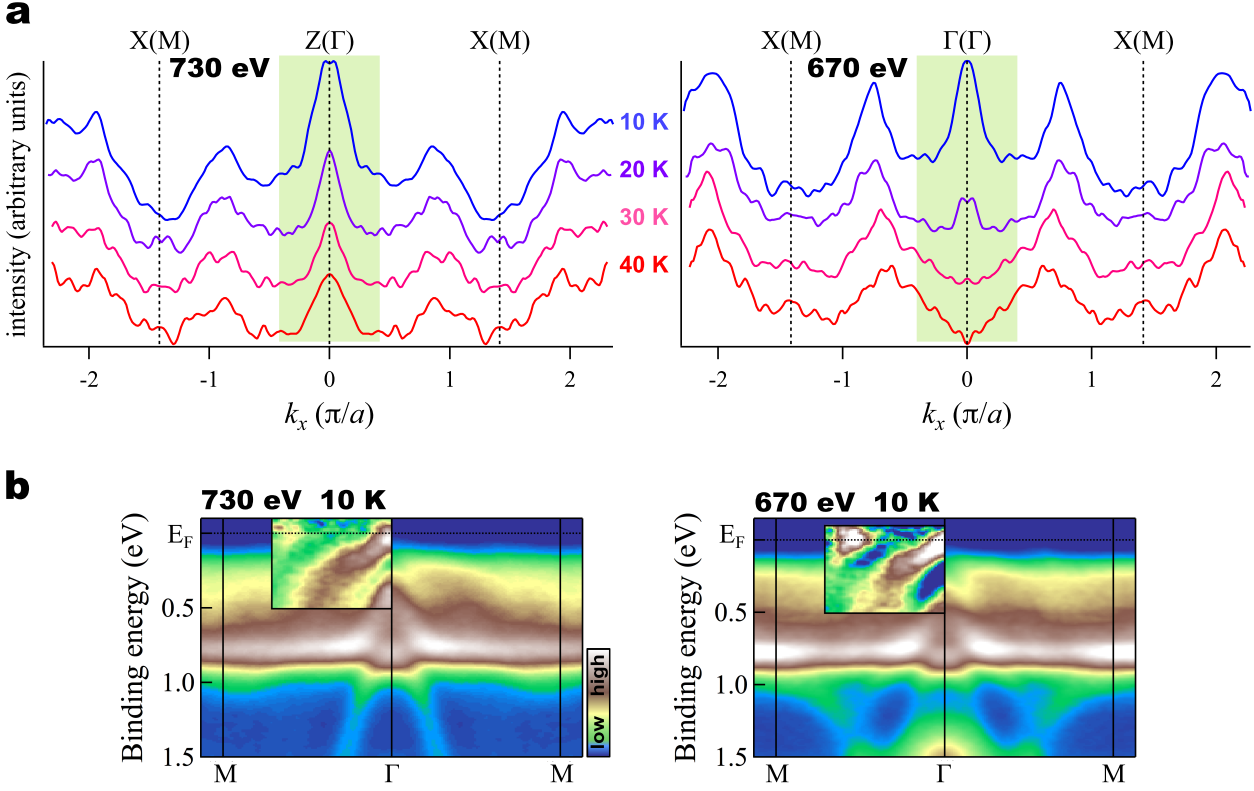


**Supplementary Figure 3:** Temperature-dependent soft X-ray ARPES MDCs and band maps. a) Symmetrized MDCs at the Fermi level from the soft X-ray ARPES dataset as a function of temperature. The chosen photoemission measurement arcs cut the Fermi surface along Γ(Γ) – X(M) for a photon energy of 670 eV in the PM (AFM) phase and along Z(Γ) – X(M) for 730 eV (compare Fig. 3 in the manuscript). For greater clarity, the intensities in each panel where shifted against each other. b) Corresponding exemplary symmetrized band maps for 10 K (AFM phase) proving the hole-like nature of the back-folded band. The datasets in the insets were normalized to the total MDC intensities to eliminate the large nondispersive emission from the Eu 4f excitations ranging from just below the Fermi level down to ~0.9 eV binding energy.

**Supplementary Note 4:**

We have focused on two photoemission measurement arcs through the Γ- and Z-point in the PM phase, which we determined to correspond to the soft X-ray photon energies hν=670 eV and 730 eV. The respective measurement arcs in **k**-space are shown in Fig. 3(b) in the main manuscript. As anticipated from the calculation, the essential observation is the existence of a hole-like band crossing the Fermi level around the Z-point, whereas it is completely absent at the Γ-point in the PM phase and appears only in the AFM phase. We can support this evidence of band back-folding further by adding momentum-energy distributions and MDCs at E_F_ shown in Supplementary Figure 3. Here we extracted the MDCs at the Fermi level along Γ(Γ) – X(M) for 670 eV in the PM (AFM) phase and along Z(Γ) – X(M) for 730 eV for temperatures of 40 K and 10 K, i.e. above and below the AFM transition temperature, respectively, and for two additional temperatures, 30 K and 20 K. The results are shown in Supplementary Fig. 3(a). In the 670 eV panel cutting through the AFM Γ-point, one can clearly see how the peak in the center, which is present in the 730 eV panel at the Z-point at all temperatures (highlighted by a green rectangle), is missing above the Néel temperature T_N_ = 24.5 K. Below the AFM transition, additional spectral weight appears at the Γ-point marking the back-folded band from the Z-point due to the Fermi surface reconstruction. The hole-like nature of this back-folded band is visible from the corresponding energy-momentum distributions presented in Supplementary Fig. 3(b) along the same high-symmetry directions as in panel Supplementary Fig. 3(a). This essential aspect of our theoretical finding is therefore supported by our measurements.

*Demonstration of the hole-like nature of the oval hole pockets*

**Supplementary Figure 4:** Energy-momentum band map along the oval hole pockets. (a) Raw band map data along the Brillouin zone cut indicated by the dashed yellow line in the inset in panel (b). Green arrows point to the Fermi vectors of the hole-like bands forming the small oval hole-like pockets, which are discussed in Fig. 4 of the manuscript as feature 3. b) Curvature of the data in panel (a) with enhanced visibility of the hole-like bands forming the oval pockets. The inset shows the combined calculated and measured Fermi surface of EuRh_2_Si_2_ presented in Fig. 4(d) in the manuscript.

**Supplementary Note 5 :**

*AFM structure and magnetic correlations in YbRh_2_Si_2_ and EuRh_2_Si_2_*

The antiferromagnetic transition at T_N_ = 70 mK in YbRh_2_Si_2_ is well established from transport, thermodynamic and magnetic measurements [4-8]. However, the antiferromagnetic structure of the Kondo lattice material YbRh_2_Si_2_ is yet unknown, the only available experimental information being on the size of the ordered moment, which seems to be extremely small, of the order of only 0.002 μB/Yb [9]. However, some information on the kind of magnetic correlation prevailing in this compound can be deduced from other properties, and this information evidences a strong similarity to the intersite correlations in EuRh_2_Si_2_, but strong differences to the intersite correlations observed in the homologues with trivalent rare earths. Thus in YbRh_2_Si_2_ there is conclusive evidence for a strong competition between FM and AFM correlations, the AFM overwhelming the FM ones only at very low temperatures [10-13]. Similarly, EuRh_2_Si_2_ orders AFM, but a tiny field of just 0.1 T applied along the easy plane is sufficient to induce an almost saturated state [14]. This implies EuRh_2_Si_2_ to be very close to the FM state, and thus indicates a competition between AFM and FM correlations similar to that in YbRh_2_Si_2_. This is in strong contrast to the systematic behavior observed in R^3+^Rh_2_Si_2_, R^3+^Co_2_Si_2_ and R^3+^Co_2_Ge_2_ compounds with trivalent magnetic rare earth R^3+^. They all show a very stable A-type AFM order, the only exception being the Kondo systems as e. g. CeRh_2_Si_2_ as well as the induced moment system PrCo_2_Ge_2_ [15, 16] where additional mechanisms lead to an instability of the local moment. Thus e.g. in GdRh_2_Si_2_ where the trivalent Gd has the same S = 7/2 local moment as the divalent Eu in EuRh_2_Si_2_, the field required to get saturation, i.e. to overcome the AFM correlations, is of the order 45 T [17], about 3 orders of magnitude larger than in the Eu system. This implies the FM state to be energetically far above the AFM state in GdRh_2_Si_2_. Thus available information on the intersite correlations in YbRh_2_Si_2_ indicate a very strong similarity to EuRh_2_Si_2_ with divalent Eu, and strong differences to homologue systems with trivalent rare earths.

**Supplementary References:**

[1] G. Zwicknagl J. Phys.: Condens. Matter **23** 094215 (2011).

[2] P. M. C. Rourke et al., Phys. Rev. Lett. **101** 237205 (2008).

[3] M. Güttler et al., Phys. Rev. B **90** 195138 (2014).

[4] J. Custers et al., Nature **424**, 524 (2003).

[5] C. Krellner et al., Phys. Rev. Lett. **102**, 196402 (2009).

[6] O. Trovarelli et al., Phys. Rev. Lett. **85**, 626 (2000).

[7] M. Brando et al., PSSB **250**, 485 (2013).

[8] H. Pfau et al., Nature 484, 493 (2012).

[9] K. Ishida et al., Phys. Rev. B **68** 184401 (2003).

[10] K. Ishida et al., Phys. Rev. Lett. **89** 107202 (2002).

[11] J. Custers et al., J. Phys.: Condens. Matter **15** S2047 (2003).

[12] C. Stock et al., Phys. Rev. Lett. **109** 127201 (2012).

[13] S. Lausberg et al., Phys. Rev. Lett. **110** 256402 (2013).

[14] S. Seiro et al., J. Phys.: Condens Matter **26** 046002 (2014).

[15] J. Leciejewicz and A. Szytula, J.M.M.M **63** & **64** 190 (1987).

[16] N. Mufti et al., Phys. Rev. B **94** 045116 (2016).

[17] K. Kliemt and C. Krellner, J. Crystal growth **419** 37 (2015).
